# PGD: Pineapple Genomics Database

**DOI:** 10.1038/s41438-018-0078-2

**Published:** 2018-09-17

**Authors:** Huimin Xu, Qingyi Yu, Yan Shi, Xiuting Hua, Haibao Tang, Long Yang, Ray Ming, Jisen Zhang

**Affiliations:** 1 Center for Genomics and Biotechnology, Haixia Institute of Science and Technology, Fujian Provincial Key Laboratory of Haixia Applied Plant Systems Biology, Fujian Agriculture and Forestry University, 350002 Fuzhou, China; 2 College of Resource and Environment, Fujian Agriculture and Forestry University, 350002 Fuzhou, China; 3 Department of Plant Pathology and Microbiology, Texas A&M AgriLife Research, Texas A&M University System, Dallas, TX 75252 USA; 4 Agricultural Big-Data Research Center and College of Plant Protection, Shandong Agricultural University, 271018 Taian, China; 5 Department of Plant Biology, University of Illinois at Urbana-Champaign, Urbana, IL 61801 USA

## Abstract

Pineapple occupies an important phylogenetic position as its reference genome is a model for studying the evolution the *Bromeliaceae* family and the crassulacean acid metabolism (CAM) photosynthesis. Here, we developed a pineapple genomics database (PGD, http://pineapple.angiosperms.org/pineapple/html/index.html) as a central online platform for storing and integrating genomic, transcriptomic, function annotation and genetic marker data for pineapple (*Ananas comosus* (L.) Merr.). The PGD currently hosts significant search tools and available datasets for researchers to study comparative genomics, gene expression, gene co-expression molecular marker, and gene annotation of *A. comosus* (L). PGD also performed a series of additional pages for a genomic browser that visualizes genomic data interactively, bulk data download, a detailed user manual, and data integration information. PGD was developed with the capacity to integrate future data resources, and will be used as a long-term and open access database to facilitate the study of the biology, distribution, and the evolution of pineapple and the relative plant species. An email-based helpdesk is also available to offer support with the website and requests of specific datasets from the research community.

## A gene database for everything pineapple

The Pineapple Genomic Database has been set up as a go-to platform for researchers to freely search for and access genetic and genomic information on the pineapple. Jisen Zhang of China’s Fujian Agriculture and Forestry University and colleagues set up the database to facilitate the study of the biology, distribution and evolution of the pineapple and its related species. The database provides information on single genes and their biological functions, gene interactions, pineapple RNA data, and information on molecular markers that can identify specific DNA sequences within the pineapple genome. Information in the database can be visualized via any device through a genome browser called JBrowse, or through a web interface, called ViroBlast, which can be used to search for nucleotide and amino acid sequence similarities.

## Introduction

Pineapple (*Ananas comosus* (L.)) is an important tropical fruit displaying crassulacean acid metabolism (CAM) with high water-use efficiency. Due to its pleasant taste and desirable nutritional characteristics, pineapple has a great economic value and industrial usage, as well as medicinal properties. Genetically, pineapple originated when the *Bromeliaceae* family separated from Poaceae early in the history of Poales offering an evolutionary reference for comparative genomics analysis of cereal genomes. In comparison with Poaceae such as rice (*Oryza sativa*), maize (*Zea mays*), sorghum (*Sorghum bicolor*)^1,2^, the genome sequence of pineapple were released much later, when the challenges of its highly heterozygous genome were recently solved and a reference genome assembled by Ming et al.^3^ The genome assembly of pineapple was published, the samples used to assemble genome were from variety F153. Because of the pivotal phylogenetic position of pineapple at the base of the Poales^3^, making its much smaller genome convenient for evolutionary studies. At present, many sequenced plant genomes have a genomics database for researchers to manage the biological information, such as TAIR (http://www.arabidopsis.org) for arabidopsis, MaizeGDB (http://www.maizeGDB.org) for maize, and RGAP (http://rice.plantbiology.msu.edu) for rice, but the current absence of such a database for pineapple, limits the ability of researchers in obtaining genomic data for pineapple.

Previously, a pineapple EST database was only developed with the bioinformatics resource hosting the fruit, root, and nematode-infected gall-expressed sequences^4^. In addition to the general plant databases, there are no genomic databases specifically designed for pineapple. However, with the advances of genomic sequencing technologies and the newly available assembled pineapple genome by Ming et al.^3^, an integrated genomics database storing gene information resources of pineapple is essential for the research community to explore the molecular biology of pineapple and the evolution of pineapple and also for *Bromeliaceae* studies. In this study, these gene sets were included in the pineapple genome database that we constructed. Here, we constructed an integrated functional genomics database for pineapple named PGD (http://pineapple.angiosperms.org/pineapple/html/index.html), in which users can readily access data using the browser and query a variety of data types from PGD including genetics, genomics, functional annotations, RNA-seq expression dataset, and molecular marker information. In addition, PGD also includes several online visualization tools such as JBrowse and ViroBlast that make it accessible from any device. The integrated gene search, dataset download function, genetic marker database, and help manual, will be improved constantly, and we hope that PGD will become a fundamental comprehensive genomics database for pineapple functional genomics research.

## Construction and content

### Data sources and processing

#### Genome assemblies and gene annotations

The pineapple reference genome was sequenced and assembled by Ming et al.^3^ using three main approaches, whole-genome shotgun sequenced with Illimina, 454 (Roche), PacBio-sequencing technologies, and BAC pools sequenced with Illumina sequencing^3^. The assembled genome included 8986 contigs with N50 of 126.5 kb and 3,133 scaffold with N50 of 11.8 Mb, respectively, accounting for 71.3% and 72.6% of assembled genome^3^ (Table 1). The transcriptome was assembled by de novo Trinity^5^ and reference-guided Trinity was constructed by PASA^6^ with the nearly full-length pineapple transcript, which was identified using BLASTP. Subsequently each transcript were trained by SNAP^7^, GENEMARK^8^, and AUGUSTUS^9^. This result was combined with gene annotation produced by MAKER, which could be considered as messenger RNA evidence^3^. A total of 27,024 gene models were obtained by MAKER annotation without redundancy, which include 24,063 complete gene models and 2,961 classified as partial.

**Table 1:** Summary of genome assembly of pineapple variety “F153” in PGD

Genome assembly	Number	N50 (kb)	Size (Mb)	Assembly (%)
Assembly scaffold	3133	11,759.3	381.9	72.6
Assembly contigs	8986	126.5	375.1	71.3

#### Gene function annotation

Based on three main protein databases, SWISS-PROT, TrEMBL, and TAIR10, the protein descriptions were defined using AHRD with optimum parameter^3^. For functional annotation, conserved domain regions and interrelated gene information, Gene Ontology (GO)^10^, and InterPro^11^ domain of the predicted pineapple proteins were annotated using InterProScan^12^ with default parameters. In addition, the relevant Kyoto Encyclopedia of Genes and Genomes (KEGG)^13^ pathway-associated pineapple protein was annotated using KOBAS^14^. The above data were publicly available and are now accessible on PGD for users (Table 2).

**Table 2:** Summary of gene annotation of pineapple variety “F153” in PGD

Gene annotation	Number	Percentage (%)
InterPro	12,762	47.2%
KEGG orthology	4229	15.6%
GO terms	6794	25.0%
Total annotated genes	13,555	50.2%

#### Genetic marker annotation: SSR, SNP, and IP markers

To develop the resources of simple sequence repeats (SSRs) of the pineapple genome, three main procedures in this execution make it possible to identify more SSRs markers. The first step is the fact that sequences of SSR loci were extracted from the pineapple genome using the customer/user Perl script. Secondly, SSR primers were designed based on 60 bp each side of target loci on the coding sequence (CDS) and genomic sequence by Primer3^15^. These primers were ultimately tested by e-PCR^16^ and the optimal results of CDS-SSR and genomic-SSR markers were selected as the reference resource to deposit in the PGD. As a result, a total of 4,629 CDS-SSR and 46,860 genomic-SSR markers were identified and made available in pineapple genome database with detailed information for the both types for users.

Regarding single-nucleotide polymorphism (SNP), a total of 89 genome resequencing *Ananas* accessions were collected, and paired-end resequencing reads were mapped to the pineapple F153 reference genome with BWA (version: 0.7.12-r1039)^17^ using the default parameters. To convert mapping results into the BAM format and to filter duplicated reads, SAMtools (vesion:1.3)^18^ and Picard package were used, respectively. The Genome Analysis Toolkit (GATK, version 3.7-0-gcfedb67)^19^ was performed to detect SNPs. The neighbor-joining tree was constructed using SNPhylo^20^ software with bootstrap value 100. We identified 7,252,423 SNPs and 923,469 indels.

To develop intron polymorphism (IP) markers, a customized Perl script was used to search IP loci in the pineapple genome, and the primer design and tests were similar to the pressure of SSR maker developments. The PGD collected 17,540 IP loci, which are also used to establish whether introns exist in the querying sequences using the IP development page.

#### Expression data

We collected and downloaded 45 RNA-seq samples from the public platform (https://de.iplantcollaborative.org/de/?type=data&folder=/iplant/home/cmwai/coge_data/Pineapple_tissue_RNAseq), these RNA-seq samples include the photosynthetic (green tip) and non-photosynthetic (white base) leaf tissue at 2-h intervals over a 24-h period during the growth period of the field from *A. comosus* cultivar MD2 (26 samples), the different leaf segments at 12:00 and 10:00 from each individual MD2 plants (12 samples), the fruits from cultivar MD2 (5 sample), the flowers (1 samples), and root (1 sample) tissues from *A. comosus* var. F153^3^. The sample information is shown in Table 3. The clean reads were obtained using Trimmomatic^21^, and were subsequently aligned to reference genomes using HISAT2 (v2.0.5)^22^. The fragments per kilobase of exon per million fragments mapped (FPKM) of the annotated genes were normalized using Cufflinks (v2.2.1) (http://cole-trapnell-lab.github.io/cufflinks/releases/v2.2.1/) with default parameters.

**Table 3:** Summary of RNA-seq samples in PGD

Species	Tissues	Collected
MD2	Leafs	Segment (S1–S6): 12:00, 22:00;
		White base/green tip: 10:00, 12:00, 13:00, 15:00, 16:00, 18:00, 20:00, 22:00, 24:00, 2:00, 4:00, 6:00, and 8:00
	Fruits	Development stage 1–6
var.F153	Developing flowers	
	Roots	

#### Gene-to-gene co-expression

In order to identify co-regulated genes in *A. comosus*, both Spearman and Pearson methods^23^ were used to calculate pairwise expression correlation co-efficiency based on RNA-seq data among 15 different tissues, which included 13 different leaves, one root, and one flower. A total of 7,228 informative genes (with FPKM >5 in at least one tissue and a variance >1) were obtained and gene pairs with absolute similarity of expression correlation >0.65 were used as the final dataset. All datasets are easily navigable and available in PGD.

#### Comparative genomics analysis

To clarify the evolutionary relationship and whole-genome duplication (WGD) events between pineapple and eight representative plant species, including *Oryza sativa*, *Vitis vinifera*, *Spirodela polyrhiza*, *Asparagus aofficinalis*, *Elaeis guineensis*, *Phoenix dactylifera*, *S. bicolor*, and *Musa acuminata*, we performed whole-genome comparative analyses. The collinear regions between pineapple and these eight plant species were visualized based on dot-plot using MCscan^24^.

### Database architecture and implementation

**Fig. 1:**
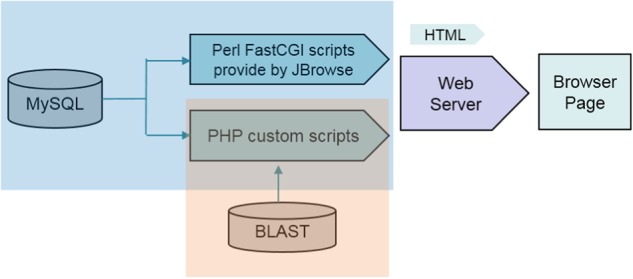
Flowchart of the Pineapple Genomics Database (PGD) sitemap

The PGD was implemented by performing a variety of several common software packages in the LINUX system, including PHP, Apache web server, MySQL database management, and Perl FastCGI. The data was processed and analyzed by the pipelines of Perl script, and bioinformation tools for interpreting biological significance. The PGD consists of some relational databases storing the processed data in MySQL. An interactive Web interface was constructed to enable users to conveniently access the PGD and obtain the information needed either for basic research applications or biological analysis through any modern browser on their devices. PHP script was implemented to transmit user query information and rapidly extracted data from MySQL databases management to generate report pages (Fig. 1). In addition, the genome visualization tool was implemented by the genetic genome browser (JBrowser)^25^. For interactive alignment of genome sequences, BLAST^26^ was performed by ViroBLAST^26^, an independent web server for flexible queries of similar nucleotide and amino acid sequences.

### Content

**Fig. 2:**
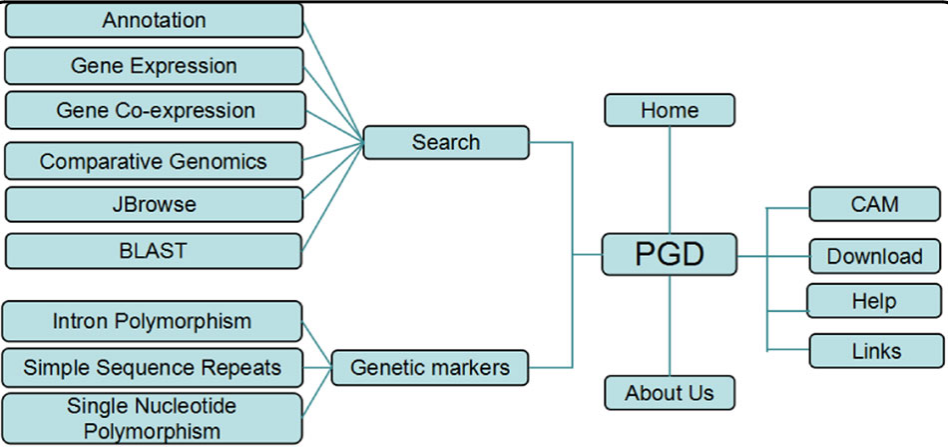
Architecture of the Pineapple Genomics Database (PGD)

The overview of the PGD’s structure is shown in Fig. 2, it consists of three main modules: search, molecular markers, and online tools. In the search module, we provided four methods to search for pineapple genes: searching specific gene information by gene properties such as gene names, term ID, and expression value based on FPKM. The relationship of gene co-expression was searched by value and gene ID. In the molecular markers module, the search page of PGD molecular marker was developed based on available public data resources containing a large number of IP, SSR, and SNP markers. Almost all the genetic markers available could be used to construct the genetic map based on microsatellites^27^ for *A. comosus* species, which can benefit both biological traits and genetic divergence studies in pineapple. In addition, online access tools provide two major functions for users, browsing gene structure by genomic regions, and searching by nucleotide and amino acid sequence similarity.

## Utility and discussion

### Search function: search genes, gene expression, and gene co-expression

The search module allows researchers to search gene information from the pineapple genome, and to do so several modes to search data are provided, including by gene ID, GO ID, KEGG ID, and InterPro ID. This module provides an interactive and user-friendly interface that also includes examples. The related term information of focused genes was provided by searching, where several hyperlinks are rendered in search results page, users can obtain interrelated core information of focused genes by clicking those hyperlinks.

**Fig. 3:**
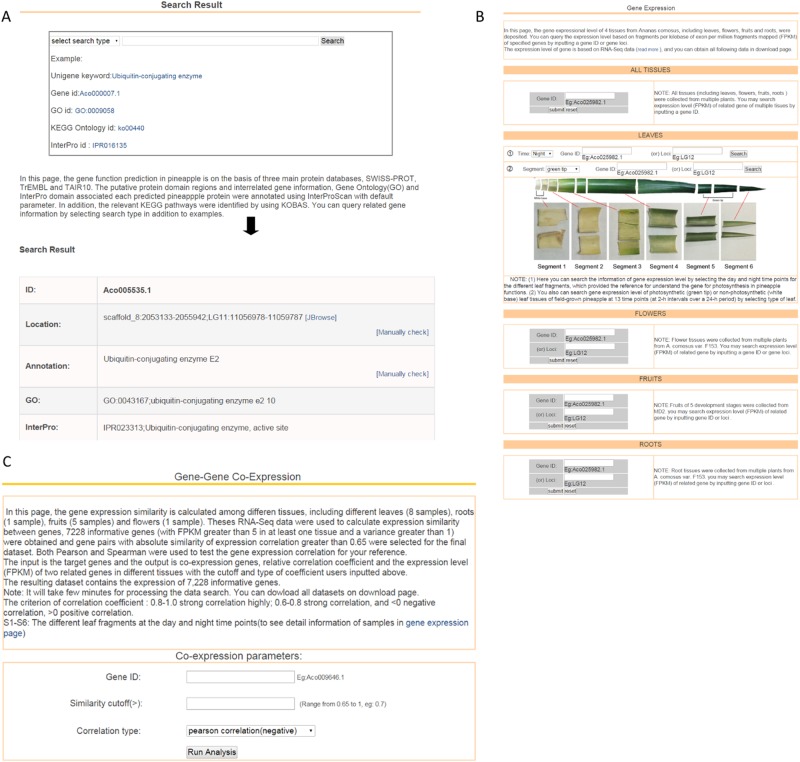
Schematic structure of the PGD. Components including gene information, overview, (**a**) annotation, (**b**) gene expression, (**c**) gene-to-gene co-expression, and their orientations are presented. Boxs represent different components

In the gene-expression page, users can query the expression level based on FPKM of specified genes by inputting a gene or gene loci. In addition, the input is the identifier of target genes and the output is co-expression genes and relative correlation coefficient with the cutoff and type of coefficient users inputted above in the gene co-expression page (Fig. 3).

### Comparative genomics

**Fig. 4:**
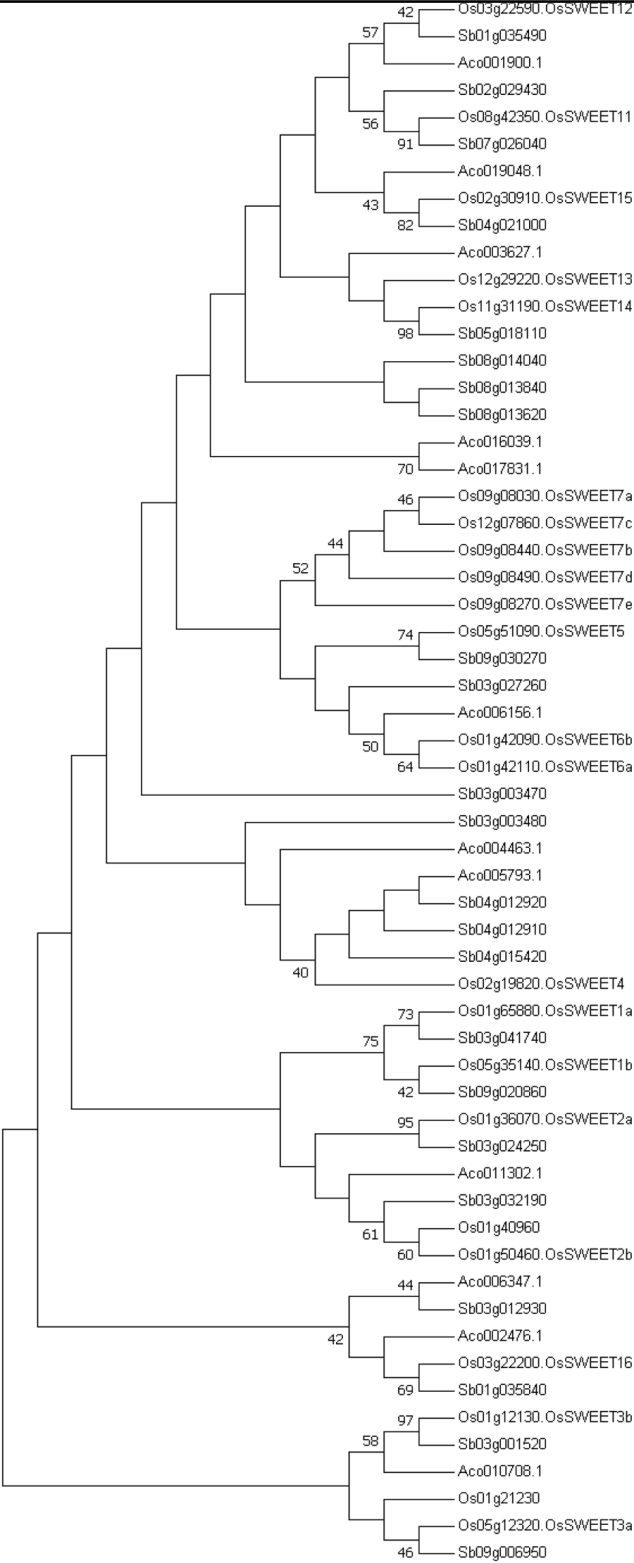
Comparison of gene structure and phylogenetic analysis of the eight members of *SWEET* gene family. Unrooted phylogenetic tree of plant *SWEET* proteins constructed using the neighbor-joining method with MEGA 7.0.26 program. Aco *Ananas comosus*, Sb *Sorghum bicolor*, Os *Oryza sativa*

Pineapples are monocotyledonous and phylogenetically related to Poaceae (including maize, wheat, rice, and sorghum), its genome is considered as a reference for comparative analysis of monocotyledons based on its well-conserved karyotype. The critical phylogenetic position on the Poales level result in the revision of the date of the cereal genome duplication event *ρ*, which was initially thought to have occurred between 9.5 and 11.5 millions years ago (MYA), This duplication event does not exist in pineapples, and the previous *σ* WGD was revised to 100–120 MYA^3^. For example, a phylogenetic analysis for the *SWEET*s (Sugar Will Eventually be Exported Transporters) gene family based on representative monocotyledon (*A. comosus*, *S. bicolor*, *O. sativa*) plant species suggested that the *SWEET* gene family is ancient and its evolutionary history can be traced in duplicated order (Fig. 4). Here, *SWEET*s expansion in Poales were also assumed to be mainly caused by *ρ* WGD, one of the pineapple *SWEET* genes (Aco003627.1) where the recent gene duplications contributed to *SWEET* expansion in rice. But in another group, the Poales plant likely retained the *SWEET*s inherited from *σ* WGD (for example: Aco004463.1), and one of the ancestors subsequently acquired *ρ* WGD in the lineages leading to rice and sorghum, which generated two *SWEET*s. The Poales lineage separated from the lineages leading to banana and the palms 100–120 million years ago^28^, the earlier *σ* WGD event occurred in Poales lineage before *ρ* WGD. Pineapple lacking the *ρ* WGD represents the closest sequenced lineage to the grasses, which makes it an excellent outgroup for grass comparative genomic studies^3^.

**Fig. 5:**
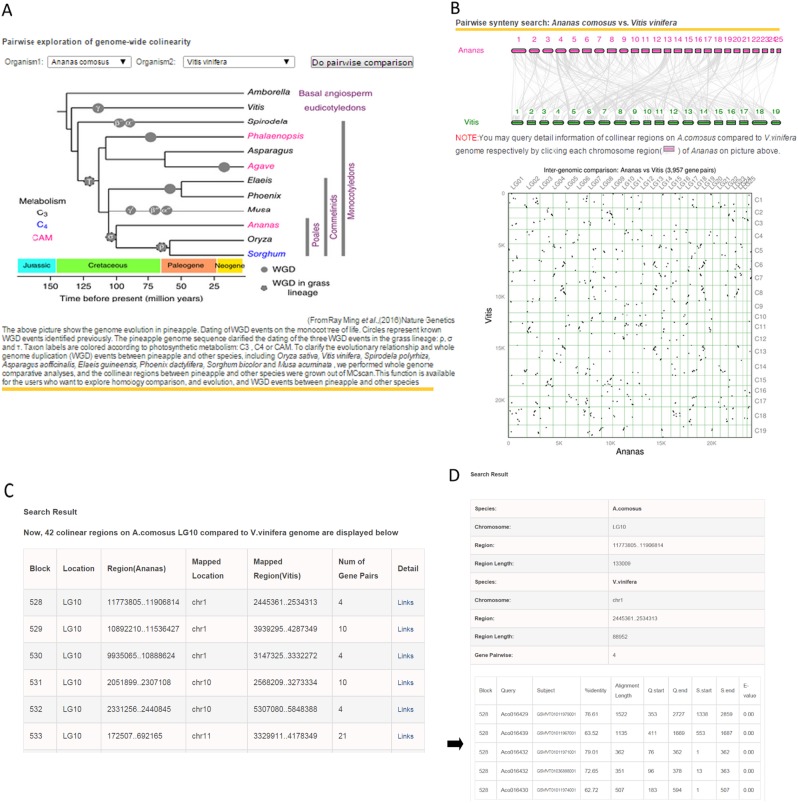
Colinear regions of *A. comosus* compared with corresponding genomes. **a** Search interface. (Phylogenetic tree cited by Ray Ming et al.). **b** Colinear regions displayed by MScan software. **c** Colinear regions list. **d** Orthologous gene pairs on colinear regions

This function is available for users who want to explore homology comparison, and evolution, and WGD events between pineapple and other species. Here, homologous regions from the pineapple genome to other species are provided by the collinear region search function, and users can also query detailed information about genes of orthologous pairs (Fig. 5). This function can help researchers to understand the collinear and evolutionary relationships between the same genome and the corresponding species.

### The molecular marker in PGD

#### SNP marker, SSR marker, and IP marker

Regardless of self-incompatibility, pineapple has high levels of heterozygosity resulting from clonal propagation. F153 had a combined heterozygosity rate of 1.89%, with 1.54% SNPs and 0.35% indels, whereas MD2 had a heterozygosity rate of 1.98%, with 1.71% SNPs and 0.27% indels. The wild *A. bracteatus* CB5 accession had a higher heterozygosity rate of 2.93%, with 2.53% SNPs and 0.40% indels^3^. About SNP from pineapple, users can query all variation type of very sequenced variety of pineapple by browsing JBrowse. PGD also provided an interactive interface for users to search molecular markers data by filling out search boxes and selecting special items. Meanwhile, there are two types of query page for CDS-SSR and genome-SSR for every species where users can obtain detailed information (e.g., start and end loci, forward sequence, reverse sequence, forward length, reverse length, forward GC, and reverse GC and so on) by clicking submit bottom on the query pages.

In addition, for IP molecular markers, the data here can be searched including: chromosome number, intron start, intron end, intron length, forward sequence, reverse sequence, forward Tm, reverse Tm, forward GC, and reverse GC.

### BLAST server and genome visualization

**Fig. 6:**
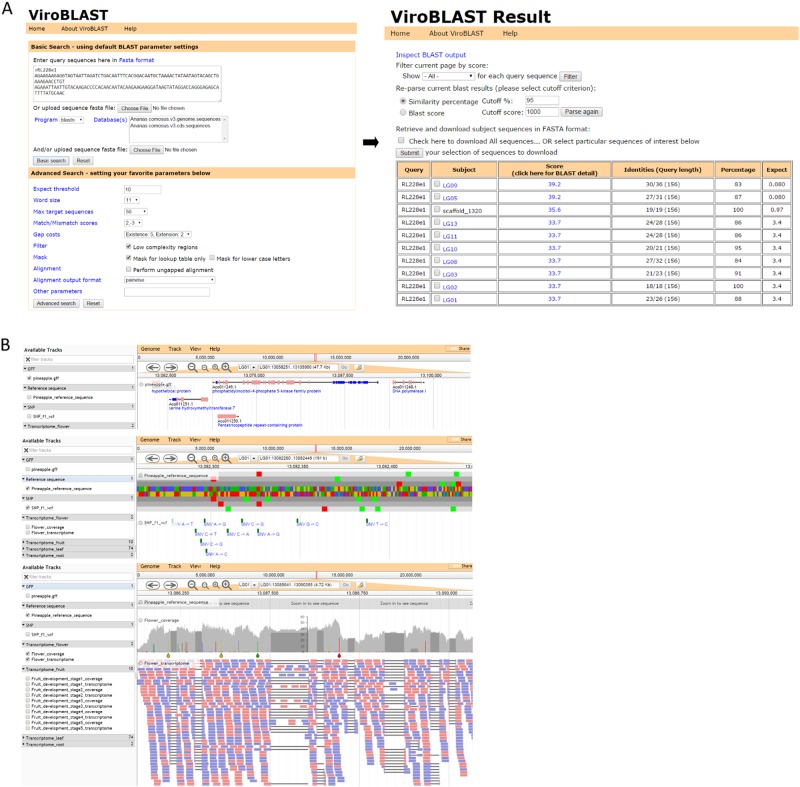
Visualization Tools Interface. **a** ViroBlast for sequence homology searches. **b** Visualize the genome and supporting data using JBrowse: the annotation and structure of our genes, SNPs, and RNA-seq data

BLAST was implemented by using ViroBlast for sequence homology searches. User can search sequences of pineapple including genomic scaffolds, coding sequences, or proteins. BLASTN, TBLASTX, and BLASTX can be conducted to search the sequences of scaffolds, unigenes, and gene CDS. In addition, users can also search against databases of protein sequences by inputting protein and nucleotide sequences within BLASTP and BLASTX, respectively. Besides, users can query protein sequences using TBLASTX, a tool of translating nucleotide sequences entered and nucleotide databases into protein sequences (Fig. 6a).

JBrowse was developed for displaying the annotation and structure of our genes, functionally annotated unigenes, SNP, and RNA-seq data. Users can query genomic scaffolds, which enables users to view distinctly the relevant information for gene annotation and structure when assessing gene models. (Fig. 6b).

### Data download, statistical information, and user manual

The download page was provided for users to download entire datasets as needed, including genomic scaffolds and predicted gene sequences in the FASTA format and gene structure in the gff3 format. The dataset of gene annotation contains gene functional descriptions, KEGG, GO, and InterPro domain, which was provided for users in a download page. Pineapple transcriptome data from leaf, the different development stages of fruits, flower, and root are available for expression information of the corresponding genes. In addition, the general statistics data of genome assemblies, gene expression, homology, gene models, gene co-expression, and genetic molecular marker in each species are displayed in interactive page readily. Additionally, we also provide a detailed user manual, including data resources, sequence processing methodology and parameters, and user documents for users in PGD, as well as manually correction documents for users generating useful and practical recommendations.

## Limitations and future development

Some functional genomics information that cannot be fully accessed in all species in PGD remain due to the limitations of current assemblies and annotations. This information includes for example, alternative spliced events and non-coding RNA genes that are not annotated in most of the varieties of pineapple.

Pineapples are monocotyledonous and phylogenetically related to Poaceae plants (including maize, wheat, rice, and sorghum) and are the best genomes for studying the gene family evolution in Poaceae and monocot genomes. In addition, PGD will explore these two directions in future developments. Due to the rapid development of genome analysis, the variety of datasets for gene expression and sequence and the structure and function of the current annotation will be improved in the future. Besides, the novel functional genomics data resources will be displayed in PGD in the future, based on the recently released public data and data from our research group with respect to pineapple, including information about non-coding RNA, and comparative genomics.

## Conclusion

We developed the Pineapple Genomic Database (PGD), which includes the large amount of set of genomic data and several online visualization tools for future research on genomics, molecular marker, and transcriptome of pineapple. Several powerful search tools were implemented, which allow users to analyze their target genes. This site aims to be the database-to-go for pineapple, thanks to intuitive search options, visualization, downloading, mining literature, and cross-species searches, providing the latest, unrestricted access to genomic data to end-users. Integrating all of these resources in a portal, and providing useful *Ananas* and comparative genomic specific resources, will help fostering a global and active *Ananas* research community and genome evolution study of Poales.

## Availability and requirements

The PGD can be freely accessed at http://pineapple.angiosperms.org/pineapple/html/index.html via the World Wide Web. A reliable data management system has been developed and all newly released information will be updated on this website. Enquiries concerning the database should be directed by email to rayming@illinois.edu or zjisen@126.com.
